# Preliminary Exploration of Metabolic Mechanisms in Copper-Exposed *Sepia esculenta* Based on Transcriptome Analysis

**DOI:** 10.3390/metabo13040471

**Published:** 2023-03-25

**Authors:** Zan Li, Lisheng Jiang, Tao Xu, Xiaokai Bao, Weijun Wang, Yanwei Feng, Jianmin Yang, Jingjun Ma

**Affiliations:** 1 School of Agriculture, Ludong University, Yantai 264025, China; 2 Yantai Laishan District Fisheries and Marine Service Station, Yantai 264003, China; 3 Shandong Marine Resource and Environment Research Institute, Yantai 265503, China; 4 Shandong Fishery Development and Resource Conservation Center, Jinan 250013, China

**Keywords:** Cu, metabolism, protein–protein interaction network, *Sepia esculenta*, transcriptome

## Abstract

As a common and high-concentration heavy metal in the ocean, Cu can induce metal toxicity and significantly affect the metabolic function of marine organisms. *Sepia esculenta* is an important economic cephalopod found along the east coast of China, the growth, movement, and reproduction of which are all affected by heavy metals. Hitherto, the specific metabolic mechanism of heavy-metal exposure in *S. esculenta* is still unclear. In this study, we identified 1131 DEGs through transcriptome analysis of larval *S. esculenta* within 24 h of Cu exposure. GO and KEGG functional enrichment analysis results indicated that Cu exposure may affect purine metabolism, protein digestion and absorption, cholesterol metabolism, and other metabolic processes in *S. esculenta* larvae. It is worth noting that in this study we explore metabolic mechanism of Cu-exposed *S. esculenta* larvae through the comprehensive analysis of protein–protein interaction network and KEGG enrichment analysis for the first time and find 20 identified key and hub genes such as CYP7A1, CYP3A11, and ABCA1. Based on their expression, we preliminarily speculate that Cu exposure may inhibit multiple metabolic processes and induce metabolic disorders. Our results lay a foundation for further understanding the metabolic mechanism of *S. esculenta* against heavy metals and provide theoretical help for *S. esculenta* artificial breeding.

## 1. Introduction

Recently, the rapid development of heavy industry, shipbuilding, metallurgy, oil extraction, and other industries has significantly increased heavy metal concentration in the ocean, especially in coastal areas, seriously damaged the marine environment and reduced the biodiversity of polluted areas [1,2,3,4,5,6,7]. Marine organisms easily accumulate heavy metals but fin them difficult to degrade [2,4]. Heavy metals enter the organism through respiration, skin penetration, being eaten, and other ways and accumulate in a large concentrating, reducing the biological development speed and movement ability and possibly inducing death [7,8]. Previous studies have shown that when the concentration is lower than about 10 μg/L, Cu promotes the growth and development of aquatic organism larvae [9,10]. As an indispensable trace element, Cu regulates ion transport, the synthesis of functional proteins, hematopoiesis, and other biological processes, and maintains the growth of organisms [11,12,13]. For instance, as the key cofactor of many biological processes and the basic metal of all living cells, Cu regulates energy metabolism, melanin synthesis, tissue growth, and other processes [14,15]. Meanwhile, Cu is an important component of Cu/ZnSOD in mollusks, which regulates biological antioxidant reaction and effectively removes active oxygen species [16,17]. However, organisms can be poisoned when the accumulation of Cu exceeds the physiological tolerance level, resulting in oxidative damage, cellular structure destruction, metabolic disorders, physiological disorders, and other negative effects [18,19,20]. In previous studies, Cu exposure was found to inhibit the energy metabolic process of *Larimichthys crocea* [21]. Chan et al. indicated that Cu stress significantly affected lipid metabolism, severely affecting lipid growth and reproduction [22]. Although metabolic mechanisms after Cu exposure have been widely studied in multiple aquatic organisms, they have been rarely studied in cephalopods with a Cu concentration in meat lower than 50 mg/kg (NY5073-2006).

As an important economic cephalopod distributed in the east coast of China, golden cuttlefish (*Sepia esculenta*) has rich nutrition and high medicinal value [23]. Because of these advantages, the *S. esculenta* has been caught in large quantities in recent years, inducing a sharp decrease in the wild population [24]. The larvae of *S. esculenta* are relatively fragile, and their growth and development are vulnerable to the impact of chemical pollutants in the ocean [25]. Previous studies have shown that Cu exposure significantly affects metabolic processes such as nucleotide metabolism and energy metabolism in mollusks [26,27]. As a result, in order to protect wild species or promote the development of artificial culture, it is necessary to explore the metabolic mechanisms of *S. esculenta* larvae exposed to Cu.

RNA-Seq explores the differences between samples at the gene level [1,3,28]. It can be used not only in model species and higher vertebrates, but has recently also been used in most mollusks, which promotes the development of mollusk biology [2,29,30]. Recently, RNA-Seq was found to be able to effectively explore the immune, metabolic and toxicological mechanisms of organisms exposed to heavy metals such as Cu, Cd, and Cr [31,32,33]. Hence, the metabolic mechanisms of Cu-exposed *S. esculenta* can be analyzed through RNA-Seq.

Thus, in our research, we use functional enrichment and protein–protein interaction (PPI) network analyses to explore key genes and signaling pathways. Among this, a comprehensive analysis of the KEGG and PPI network is first used to study metabolic mechanisms of Cu-exposed *S. esculenta* larvae. The results show that Cu may inhibit the metabolism of protein, lipid, and cholesterol in *S. esculenta* larvae, thus affecting the development of cells and tissues, and that they may inhibit the production and transport of energy to inhibit larval growth. Our results have deepened the understanding of the metabolic mechanism of invertebrates exposed to Cu and promoted the development of marine environmental toxicology.

## 2. Material and Methods

### 2.1. S. esculenta Larvae and Exposure

An adult collected from the Qingdao coast was temporarily raised for a week, and eggs were laid and collected in flowing seawater at a temperature of 21.5 ± 1.5 °C and a salinity of 30.4 ± 0.3. About four weeks later, larvae were hatched and divided into the control group (C) and the Cu-exposed group (Cu). According to the relevant research results from our laboratory on *S. esculenta* [34,35], 50 μg/L of Cu was produced by dissolution of CuCl_2_ × H_2_O_2_ powder with 99% AR was used to expose larvae. We collected larvae at 0 h (C_0 h), 4 h (C_4 h and Cu_4 h), and 24 h (C_24 h and Cu_24 h). The larvae samples were loaded into the sterile tubes after quick freezing in liquid nitrogen and then stored in liquid nitrogen.

### 2.2. Sequencing and Transcriptome Analysis

We used TRI reagent to extract total RNA. The equal molar masses of RNA from three randomly selected larvae in each group were mixed into a replicate, and the process was repeated three times. The above three replicates were used for transcriptome library construction. NEBNext^®^ Ultra™ RNA Library Prep Kit for Illumina^®^ (San Diego, CA, USA) was used to construct sample libraries. First, a sample of mRNA was obtained by purifying total RNA using poly-T oligo-attached magnetic beads. The mRNA was then smashed into fragments in a fragmentation buffer. Next, the first-strand cDNA was synthesized using random hexamers, and the second-strand cDNA was synthesized in a buffer containing dNTPs, DNA polymerase I, and RNase H. Subsequently, the cDNA was purified, end repaired, linked to poly-A, and ligated to an adaptor. Finally, cDNA was amplified using PCR, and AMPure XP beads were used to purify the products. Larval samples were sequenced by an Illumina NovaSeq 6000 (Illumina, San Diego, CA, USA).

Reads containing adapters, more than 10% unknown nucleotides, and more than 50% of Q-value ≤ 20 bases were removed. After removing low-quality sequences and performing mapping, DEGs were identified using DESeq2 [36] with *p* value ≤ 0.05 and fold change ≥ 1.5. During the screening process, the identification results were revised for multiple testing with a parameter FDR < 0.01.

### 2.3. Gene Function Identify and Network Construction

DAVID v6.8 was used to enrich DEGs into the GO terms and KEGG signaling pathways [37]. The reference genome was used as the background gene set and DEGs were used as a validation set to analyze the differences in metabolic mechanisms within 24 h of Cu exposure. Then, DEGs were enriched into KEGG pathways and GO terms of biological process, molecular function, and cellular component (*p* value ≤ 0.05). Finally, significantly enriched metabolism-related terms and pathways were identified to explore *S. esculenta* larval metabolic mechanisms.

DEGs, enriched in significant metabolism-related KEGG pathways, were used to construct a PPI network using STRING v11.0 with default parameters [38]. Briefly, protein sequences were initially supplied to STRING and mapped to its database. Then, proteins were identified and used to construct the network based on their functions. Finally, the parameters were adjusted and the proteins that did not interact with other proteins were removed. Twenty DEGs with high protein interaction numbers were selected and regarded as key genes for the regulating metabolic processes of *S. esculenta* larvae. Three DEGs with the highest protein interaction numbers were defined as the hub genes most likely to regulate larval metabolism.

### 2.4. Quantitative RT-PCR Assay

qRT-PCR was used for verifying the accuracy of RNA-Seq [39]. We designed gene-specific primers using Primer Premier 5.0. Table S1 shows their primer sequences. Before validation, we screened three reference genes, including *GAPDH*, *β-actin*, and *18S*, and determined the stability of their expression level. Finally, we used the most stable *β-actin* for qRT-PCR.

### 2.5. Statistical Analysis

The relative mRNA abundance of key genes verified by qRT-PCR was calculated with the 2^−ΔΔCT^ method [40]. Significance analysis was performed via *t* test. Letters a, b, and c indicate significant difference.

## 3. Results

### 3.1. Sequencing Quality

Sequencing results show that an average of 44,016,008 raw reads and 43,510,849 clean reads are sequenced. The averages of Q20 and Q30 are 97.42% and 92.98%, respectively, and the average of GC of clean reads is 39.76% (Table S2). Raw sequencing reads were submitted to the Sequence Read Archive in NCBI. The BioProject accession number was PRJNA844162; and the BioSample accession numbers were SAMN28794853, SAMN28794854, SAMN28794855, SAMN28794856, and SAMN28794857.

### 3.2. DEGs Expression

**Figure 1:**
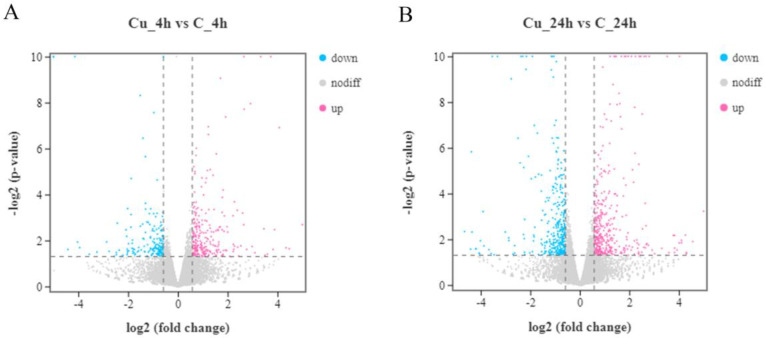
Expression difference of DEGs. (**A**) DEGs expression distribution at 4 h exposure. (**B**) expression distribution of DEGs at 24 h exposure.

**Figure 2:**
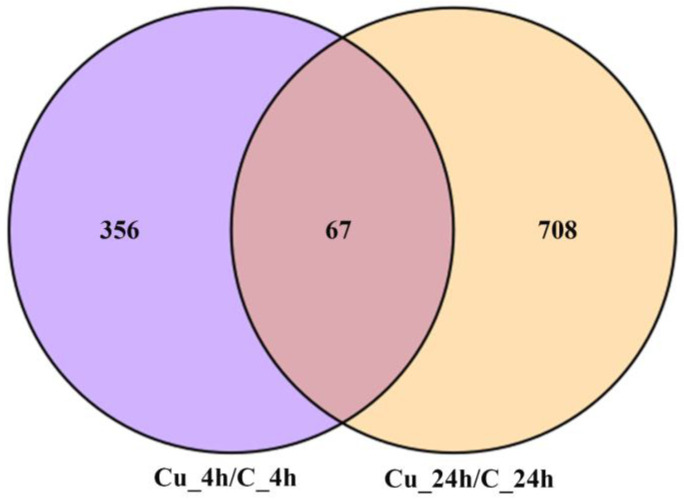
DEG distributions between two time points. Different colors represent different DEGs expression distribution.

**Figure 3:**
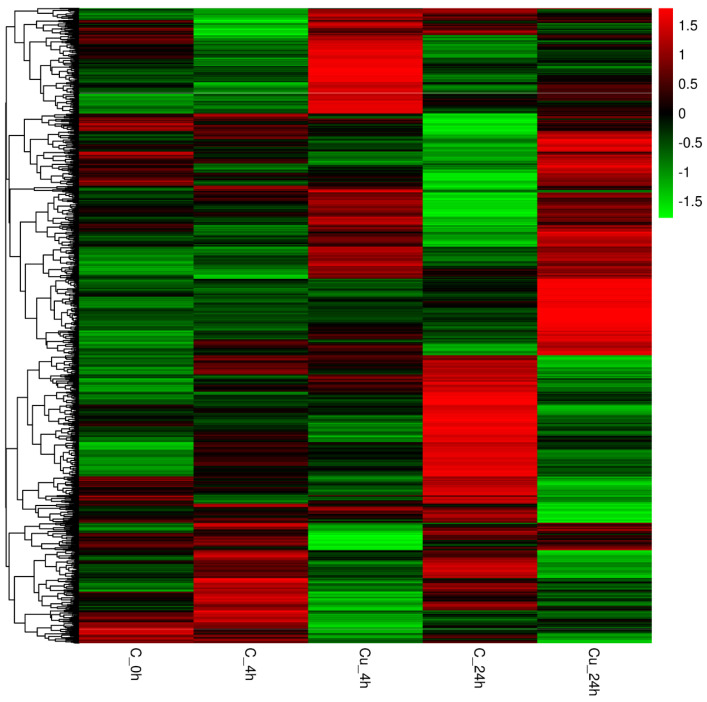
Expression clustering of DEGs. A row indicates expressions of a DEG in each group; each column represents the expressions amount of all DEGs in a group.

After the differential expression analysis, 423 (256 up-regulated and 167 down-regulated) and 775 (408 up-regulated and 367 down-regulated) DEGs were identified at 4 and 24 h, respectively (Figure 1). Figure 2 shows that a total of 1131 DEGs expression difference within 24 h exposure, and 67 DEGs are differentially expressed at two time points. DEGs expression distribution is shown in the heatmap (Figure 3).

### 3.3. DEGs Functions

**Figure 4:**
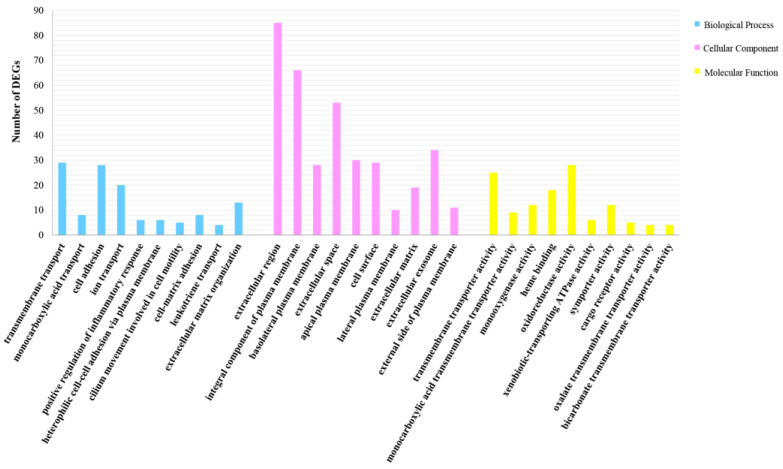
Top 10 significant GO terms. Ordinate indicates DEG numbers; abscissa stands for specific terms.

**Figure 5:**
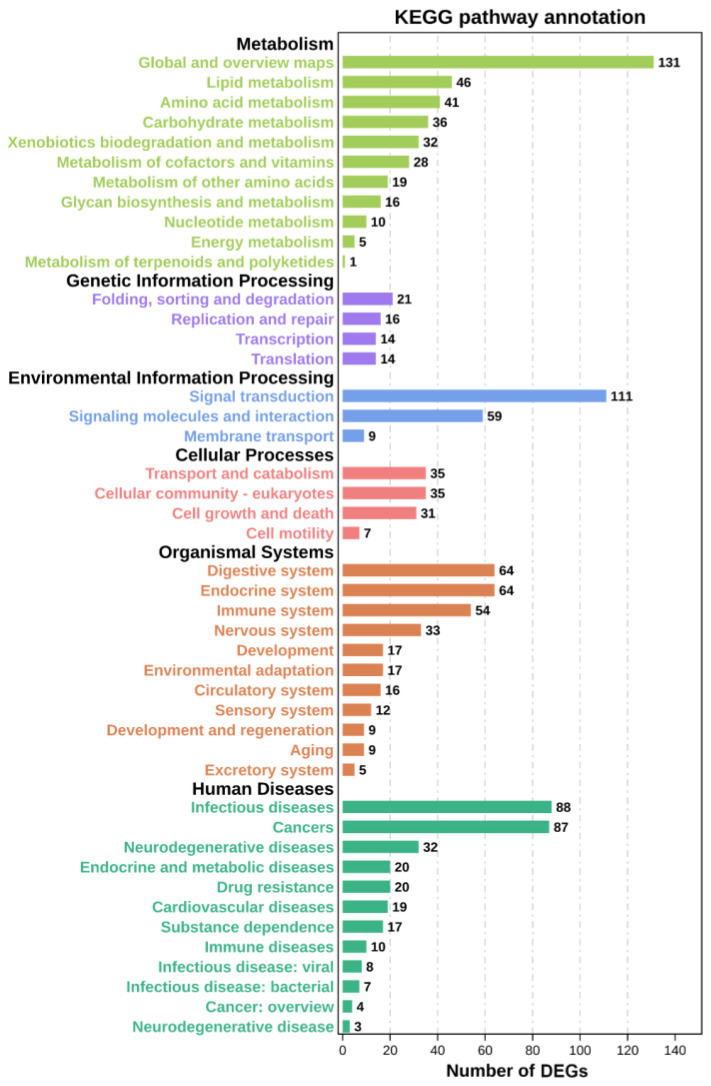
Level-2 KEGG signaling pathways statistics.

A total of 95 significant GO terms are enriched in this study (Figure 4). Among them, ion transport, arachidonic acid metabolic process, glucose-6-phosphate transport, glucose homeostasis, monocarboxylic acid transport, and other significant terms are important for regulating metabolism. The down-regulation of DEGs enriched in these terms suggests that Cu inhibits these metabolic processes. Meanwhile, metabolism-related level-2 KEGG signaling pathways, such as the glutathione metabolism, glycerolipid metabolism, and lipid metabolism pathways, are enriched (Figure 5), and Cu inhibits them within 24 h of exposure. A total of 20 level-3 KEGG signaling pathways (Table 1) are also enriched, such as the protein digestion and absorption signaling pathway, purine metabolism signaling pathway, cholesterol metabolism signaling pathway, PI3K-Akt signaling pathway, renin secretion signaling pathway, and ECM–receptor interaction signaling pathway. Among them, the inhibition of the PI3K-Akt signaling pathway, renin secretion signaling pathway, and ECM–receptor interaction signaling pathway indicates that Cu destroys the structure of the extracellular matrix and affects the production and transport of energy. In addition, Cu inhibits the protein digestion and absorption process, cholesterol metabolism process, and purine metabolism process.

### 3.4. Key and Hub Genes Identify and Verification

**Figure 6:**
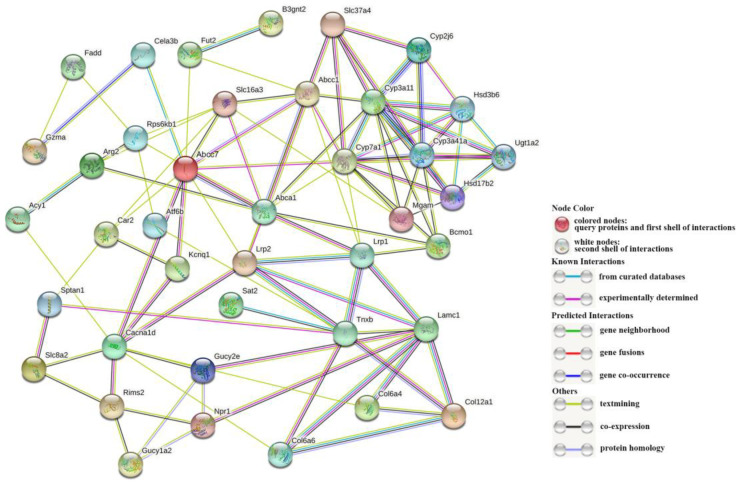
The PPI network. The dots stand for proteins, and the connection represents the interaction between genes.

In our research, 40 DEGs in Table 1 are used for PPI network construction (Figure 6). Table S3 shows relevant parameters. Among the network, three hub genes interacting with the most genes or involved in the most pathways in Table 2, including CYP7A1, CYP3A11, and ABCA1, were identified. The above three genes were up-regulated after Cu exposure, suggesting that some genes are activated and significantly expressed to maintain metabolic stability after Cu exposure. Additionally, 17 key genes with higher protein interaction numbers or higher KEGG pathway participation numbers were identified at the same time (Table 2). The close relationship between the functions of these genes indicates that Cu may induce changes in part of metabolic networks and affect the metabolic process of *S. esculenta* larvae in many ways.

**Figure 7:**
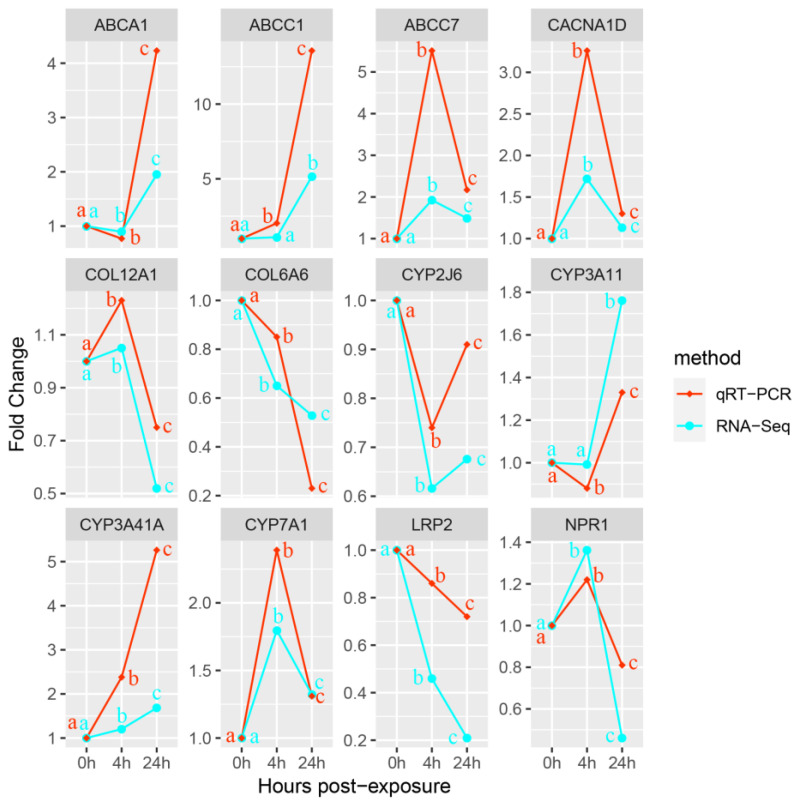
Gene expression verification (qRT-PCR). The abscissa represents Cu exposure time; the ordinate stands for fold change. a, b, and c represent significance of changes in the expression of key genes within 24 h of Cu exposure.

The qRT-PCR result indicates that DEGs in Table 2 were single products. The consistent expression trend of qRT-PCR and RNA-Seq suggests that the results of RNA-Seq are accurate (Figure 7).

## 4. Discussion

### 4.1. Metabolic Differences in Mollusk Exposed to Cu

Cu has a high accumulation capacity in marine organisms and induces changes in metabolic function when it accumulates to a certain level [20]. Alamo et al. found that higher Cu bioaccumulation might inhibit fatty acid metabolism in scallops [41], which is consistent with the significant down-regulation of DEGs enriched in the arachidonic acid metabolic process term and linoleic acid metabolism signaling pathway in this study. This result suggests that the metabolism of fatty acid in larvae may be inhibited. Most DEGs enriched by glucose-6-phosphate transport term, glucose homeostasis term, and other metabolic terms are down-regulated, a result which is consistent with previous studies on clams and oysters [26,27], indicating that energy and nucleotide metabolisms may be inhibited. The down-regulation of genes in purine metabolism signaling pathway is consistent with the results found by Zhou et al. in *Bathymodiolus platifrons* [27]. At present, the cognition of the effect of heavy metals on the metabolism mechanism of cephalopods is still in its infancy. This study explores the metabolism of Cu-exposed *S. esculenta* larvae and promotes the development of research on the metabolism mechanism of cephalopods.

### 4.2. Gene Functions Based on GO

The results of GO enrichment analysis suggest that ion transport, monocarboxylic acid, and other processes are significantly inhibited in Cu-exposed *S. esculenta* larvae. Previous studies have shown that the enrichment of th ion transport term indicated that Cu exposure might inhibit the transport of Ca^2+^, Na^+^, and other metal ions, thereby disrupting ion homeostasis and inhibiting multiple cellular metabolic processes [42]. Meanwhile, the down-regulation of DEGs enriched in monocarboxylic acid transport term suggested that metabolic processes of monocarboxylic acids such as lactate and pyruvate might be inhibited after Cu exposure [43].

### 4.3. KEGG Functional Enrichment Analysis

Twenty significant KEGG pathways are enriched, and some of them have been reported to regulate the expression of metabolic genes and the affected metabolic processes such as lipid metabolism and energy metabolism, indicating that the energy supply system and tissue growth of *S. esculenta* larvae exposed to Cu may be significantly affected [44,45]. Among them, purine metabolism, cholesterol metabolism, and protein digestion and absorption can be identified as the most likely signaling pathways to regulate the metabolism of Cu-exposed *S. esculenta*, and their specific functions are deeply explored.

#### 4.3.1. Purine Metabolism

Purines are the most abundant metabolites and are present in all organisms. They are essential components of DNA and RNA and play integral roles in cellular processes [46]. For example, they can provide energy and cofactors for proliferation, differentiation, survival, and other cellular processes [47]. Purine metabolism is an important metabolic process in regulating the synthesis and decomposition of purines and maintains the stability of purine content [46]. In addition to cellular processes, purine metabolism regulates energy metabolism and signal transduction [48]. The expression levels of most genes enriched in the purine metabolism signaling pathway were significantly down-regulated compared to control groups within 24 h of Cu exposure in this study. This result is consistent with those found by Zhou et al. in a study on clams [27]. Additionally, Hadizadeh et al. found that Cu inhibited purine metabolism by inhibiting the expression of the key enzyme, xanthine oxidase [49]. Based on previous research results and the down-regulation of GUCY1A2 and GUCY2E, we preliminarily speculate that Cu may induce purine metabolism disorder by inhibiting the expression of guanylate cyclase, thus inhibiting the growth of cells and tissues.

#### 4.3.2. Protein Digestion and Absorption

Proteins are biological macromolecules necessary for biological life activities and these play key roles in carrier transport, enzyme catalysis, and other physiological processes [50]. Meanwhile, proteins can participate in and regulate biological growth metabolism, energy metabolism, and other metabolic processes [51,52]. Proteins can be hydrolyzed into amino acids by specific proteases and transported into tissues and organs, thereby promoting cell and tissue growth [53]. This process is beneficial to maintaining the stability of biological metabolic functions, thus promoting biological growth [53,54]. In this study, we found that two of five genes enriched in protein digestion and absorption signaling pathway belong to collagen families such as COL6A6 and COL12A1. Additionally, these two genes have been identified as key genes that may regulate metabolic processes of *S. esculenta* larvae after Cu exposure. In previous studies, Hynes and Ricard-Blum found that COL6A6 and COL12A1 play significant parts in growth metabolism regulation and promote the growth, proliferation, migration, and differentiation of cells by binding to receptors [55]. Their expression levels are significantly down-regulated in this study, indicating that Cu exposure may inhibit some cellular functions and inhibit growth and metabolic processes. In conclusion, we preliminarily speculate that Cu exposure might inhibit the protein digestion and absorption process of *S. esculenta* larvae and affect cell growth, proliferation, and other cellular processes, thereby inhibiting the growth and development of larvae.

#### 4.3.3. Cholesterol Metabolism

Cholesterol is the most abundant steroid of the compounds present in various tissues [56]. It plays a key part in the synthesis of the cell membrane and regulates the metabolism of bile acid and vitamin D [57]. At the same time, it regulates the metabolism of organisms, which promotes the synthesis and release of hormones in order to regulate metabolic processes of fats, carbohydrates, and proteins [58]. Cholesterol metabolism maintains cholesterol homeostasis by regulating cholesterol synthesis and conversion, thereby maintaining the stability of cellular functions and metabolic processes [59]. Previous studies have shown that LRP1 and LRP2, as key regulators in cholesterol metabolism, play significant parts in promoting cholesterol synthesis and maintaining cholesterol homeostasis [60]. Both genes are enriched in the cholesterol metabolic signaling pathway in this study and down-regulated after Cu exposure, suggesting that Cu exposure inhibits cholesterol metabolism, which is consistent with the results found by Engle et al. [61]. Cu might disrupt cholesterol homeostasis and inhibit *S. esculenta* larval metabolic processes such as lipid and protein metabolism, thus inhibiting the synthesis, growth, and development of tissues and organs.

### 4.4. Hub Genes Functional Analysis

CYP7A1, CYP3A11, and ABCA1 are identified as hub genes in this study. They might play significant roles in regulating the larval metabolism of Cu-exposed *S. esculenta*. CYP7A1 and CYP3A11 were significant members of the cytochrome P450 (CYP) family. Based on previous research results, the CYP family has been identified as a core protein family that exists in various biological tissues to resist environmental stress [62]. CYPs were present in almost all eukaryotes. They played significant roles in cellular metabolism and maintained the cellular homeostasis of organisms [63]. For instance, they regulated multiple metabolic processes such as vitamin metabolism, lipid metabolism, and cholesterol metabolism and promoted biological growth and development [64]. Furthermore, they were involved in and regulated the metabolic processes of environmental pollutants and carcinogens and metabolized toxic substances into non-toxic or excretory substances, thus promoting the detoxification reaction [62,64]. Based on previous studies, both genes have been found regulating bile acid metabolism, cholesterol metabolism, brucine metabolism, and other metabolic processes in mammals [65,66]. However, hitherto, they have been rarely studied in mollusks, especially cephalopods, and their functions in mollusks remain unclear. In this study, CYP7A1 and CYP3A11 were up-regulated after Cu exposure. We preliminary speculate that they might have promoted *S. esculenta* larval metabolic processes such as cholesterol metabolism and lipid metabolism and have induced detoxification responses against Cu stress. Cholesterol homeostasis was critical for maintaining normal cellular processes, and excess cholesterol would inhibit cell growth and survival [67]. Previous study has shown that cholesterol efflux was currently the only way to remove excess cholesterol from cells [67,68]. ABCA1 was a significant transporter regulating this metabolic process, which mediated the transport of free cholesterol and phospholipids in cells to maintain intracellular cholesterol balance [68]. Meanwhile, ABCA1 played a key part in lipid metabolism and the regulation of apolipoprotein, which maintained lipid homeostasis [69]. ABCA1 was significantly up-regulated after slight down-regulation in this study, and Cu exposure was speculated to disrupt cholesterol balance. We initially speculated that ABCA1 might maintain cellular cholesterol homeostasis and normal function by inducing cholesterol transport. In conclusion, these three genes might play significant metabolic functions after Cu exposure, such as promoting cholesterol metabolism and lipid metabolism to maintain cellular homeostasis. At present, the metabolic functions of these genes in Cu-exposed *S. esculenta* have not been studied and need to be explored in subsequent experiences.

### 4.5. Other Key DEGs and Pathways Analyses

Unexplored key genes and signaling pathways also play significant roles in regulating metabolism. For instance, two other identified key genes of CYP family, CYP3A41A and CYP2J6, were identified in previous studies as regulating lipid metabolism [63,70,71]. Additionally, ABC transporters’ signaling pathway has been identified as regulating cholesterol synthesis and transport and regulating cholesterol metabolism [72,73]. These results further illustrate that *S. esculenta* larval metabolism has been affected by Cu. Hitherto, metabolic functions of genes and signaling pathways identified in *S. esculenta* larvae exposed to Cu have been unclear and thus now require further exploration.

## 5. Conclusions

The identification of a large number of DEGs indicated that Cu exposure might affect larval life processes. The results of functional enrichment and PPI network analyses suggested that lipid metabolism, cholesterol metabolism, and other metabolic processes of Cu-exposed *S. esculenta* might be inhibited. In conclusion, Cu exposure might induce metabolic disorders and inhibit the growth and development of larvae, and the results laid a foundation for furthering the understanding of cephalopod metabolism after heavy-metal exposure.

## Figures and Tables

**Table 1:** Metabolic pathways statistics.

Pathways	Number of DEGs
ABC transporters	3
Aldosterone synthesis and secretion	3
Apoptosis	4
Carbohydrate digestion and absorption	2
cGMP-PKG signaling pathway	4
Cholesterol metabolism	4
Cortisol synthesis and secretion	2
ECM–receptor interaction	3
GABAergic synapse	2
Insulin secretion	3
Linoleic acid metabolism	3
Metabolic pathways	7
PI3K-Akt signaling pathway	4
Protein digestion and absorption	6
Purine metabolism	3
Renin secretion	3
Retinol metabolism	6
Steroid hormone biosynthesis	6
Thyroid hormone synthesis	2
Vascular smooth muscle contraction	3

**Table 2:** Summary of key DEGs.

Gene Name (Abbreviation)	Gene Name (Official Full Name)	Number of Protein–Protein Interactions	Number of KEGG Signaling Pathways
*CYP7A1*	cytochrome P450, family 7, subfamily a, polypeptide 1	12	2
*CYP3A11*	cytochrome P450, family 3, subfamily a, polypeptide 11	11	3
*ABCA1*	ATP binding cassette subfamily A member 1	10	2
*ABCC7*	ATP binding cassette subfamily C member 7	10	1
*CACNA1D*	calcium voltage-gated channel subunit alpha1 D	9	7
*CYP3A41A*	cytochrome P450, family 3, subfamily a, polypeptide 41A	9	3
*ABCC1*	ATP binding cassette subfamily C member 1	9	1
*LRP2*	low density lipoprotein receptor-related protein 2	7	2
*NPR1*	natriuretic peptide receptor 1	4	5
*COL12A1*	collagen, type XII, alpha 1	4	2
*COL6A6*	collagen, type VI, alpha 6	4	2
*CYP2J6*	cytochrome P450, family 2, subfamily j, polypeptide 6	4	1

## Data Availability

The data presented in the study are deposited in the NCBI repository, accession number SRR19578100, SRR19578101, SRR19578102, SRR19578103, SRR19578104, SRR19578105, SRR19578106, SRR19578107, SRR19578108, SRR19578109, SRR19578110, SRR19578111, SRR19578112, SRR19578113, SRR19578114 at the following link: https://www.ncbi.nlm.nih.gov/Traces/study/?acc=PRJNA844162&o=acc_s%3Aa (accessed on 19 October 2022).

## Supplementary Materials

The following supporting information can be downloaded at: https://www.mdpi.com/article/10.3390/metabo13040471/s1, Table S1: List of primers used for qRT-PCR validation; Table S2: Sequencing quality and mapping results; Table S3: Summary of PPI network.
