# Supplementary material for: Preliminary Exploration of Metabolic Mechanisms in Copper-Exposed Sepia esculenta Based on Transcriptome Analysis
Source: Metabolites. 2023 Mar 25;13(4):471. doi: 10.3390/metabo13040471 (PMC10141105; doi:10.3390/metabo13040471)
Supplement: Supplementary file 1 [file metabolites-13-00471-s001.zip › Table S1.pdf]

Table S1 List of primers used for qRT-PCR validation.

Gene name	Forward primer (5'-3')	TM (°C)	Reverse primer (5'-3')	TM (°C)	Amplicon length (bp)
CYP7A1	CTCACTTTCGGTACCCTTTG	60	CGTGACCGTGGTAGTTAATG	60	142
CYP3A11	GCAAACACTCTGTCCTTCTT	60	GGTCAGCTTCTGCACATTAT	60	129
ABCA1	GAAAGCCTGAGCCGTAATC	60	AGCGTAGAAGGCCAGTAA	60	111
ABCC7	CCTGGTCAGTCGTTTCTTATC	60	GAGCCATCTTCAGCAATCTC	60	125
CACNA1D	GCAGATGTTTGGCAGAATAG	59	CTGTGGCAGATCGGAATAA	59	107
CYP3A41A	GCTGATACAGCTGATACGATG	60	AAGAGCTGTTGGCTTTGG	60	105
ABCC1	GTTTGGCTGCGAAATTGG	60	TCCAAGAGCACCGTAGAT	60	114
LRP2	AACCTCCCGGACATTGTA	60	GTAGGGCACAGTCTCATTTC	60	131
NPR1	CAACTGTGTGGTGGATAGTC	60	GGTGCCGTCCAAAGTAAT	60	132
COL12A1	AACTACCTGCTCCCATACA	60	GAGGGTTTCTTGTCCATCAG	60	110
COL6A6	TGAGACACGTGGCTATACT	60	TGATGGCCGATACCAATAAC	60	121
CYP2J6	TCGAGAGGAGATCCGAAAG	60	ACAGCAACTGAGGCAATC	60	120
