# Supplementary material for: Preliminary Exploration of Metabolic Mechanisms in Copper-Exposed Sepia esculenta Based on Transcriptome Analysis
Source: Metabolites. 2023 Mar 25;13(4):471. doi: 10.3390/metabo13040471 (PMC10141105; doi:10.3390/metabo13040471)
Supplement: Supplementary file 1 [file metabolites-13-00471-s001.zip › Table S2.pdf]

Table S2 Sequencing quality and mapping results.

Samples	Raw reads	Clean reads	Q20 (%)	Q30 (%)	GC (%)	Mapping rate (%)
C_0h_1	44,822,088	44,401,358	97.42	93.02	39.89	87.71
C_0h_2	46,604,268	46,067,346	97.39	92.97	38.64	87.25
C_0h_3	42,199,716	41,745,596	97.08	92.31	39.10	86.13
C_4h_1	42,594,570	42,050,900	97.56	93.35	39.79	88.28
C_4h_2	45,122,216	44,583,624	97.37	92.89	40.01	87.80
C_4h_3	43,910,186	43,339,204	97.44	93.00	39.72	87.67
Cu_4h_1	43,979,834	43,386,214	97.41	92.95	40.01	87.99
Cu_4h_2	43,743,466	43,329,698	97.47	93.04	39.84	88.14
Cu_4h_3	45,143,934	44,644,632	97.41	92.94	39.86	88.24
C_24h_1	45,374,672	44,918,056	97.51	93.17	39.82	88.32
C_24h_2	40,894,638	40,402,580	97.59	93.30	38.92	88.10
C_24h_3	42,664,646	42,060,564	97.65	93.45	38.74	87.88
Cu_24h_1	43,299,278	42,842,716	97.46	93.06	39.73	87.31
Cu_24h_2	44,860,386	44,400,024	96.56	93.22	39.91	88.31
Cu_24h_3	45,026,224	44,490,218	97.23	92.94	39.95	87.63
