# Supplementary material for: Preliminary Exploration of Metabolic Mechanisms in Copper-Exposed Sepia esculenta Based on Transcriptome Analysis
Source: Metabolites. 2023 Mar 25;13(4):471. doi: 10.3390/metabo13040471 (PMC10141105; doi:10.3390/metabo13040471)
Supplement: Supplementary file 1 [file metabolites-13-00471-s001.zip › Table S3.pdf]

Table S3 Summary of PPI network.

Network statistics	
Number of nodes	40
Number of edges	103
Average node degree	5.15
Clustering coefficient	0.441
Expected number of edges	48
PPI enrichment *P*-value	2.36E-12
